# Platform trial for off-label oncology drugs using comprehensive genomic profiling under the universal public healthcare system: the BELIEVE trial

**DOI:** 10.1007/s10147-023-02439-2

**Published:** 2023-12-19

**Authors:** Sae Ishimaru, Tatsunori Shimoi, Kuniko Sunami, Miho Nakajima, Yayoi Ando, Natsuko Okita, Kenichi Nakamura, Taro Shibata, Yasuhiro Fujiwara, Noboru Yamamoto

**Affiliations:** 1 Research Management Division, Clinical Research Support Office, National Cancer Center Hospital, Tokyo, Japan; 2 Department of Medical Oncology, National Cancer Center Hospital, Tokyo, Japan; 3 Department of Laboratory Medicine, National Cancer Center Hospital, Tokyo, Japan; 4 Department of Pediatric Oncology, National Cancer Center Hospital, Tokyo, Japan; 5 Department of International Clinical Development/Clinical Research Support Office, National Cancer Center Hospital, Tokyo, Japan; 6 Biostatistics Division, Center for Research Administration and Support, National Cancer Center, Tokyo, Japan; 7 Department of Experimental Therapeutics, National Cancer Center Hospital, 5-1-1 Tsukiji, Chuo-ku, Tokyo, 104-0045 Japan

**Keywords:** Genomic medicine, Comprehensive genomic profiling, Patient-proposed healthcare system, Molecular targeted therapy, Umbrella or basket-type trial

## Abstract

**Background:**

Precision medicine has transformed cancer treatment by focusing on personalized approaches based on genomic abnormalities. However, comprehensive genomic profiling (CGP) and access to targeted therapies are limited in Japan. This study investigates the BELIEVE trial, which aims to improve drug accessibility for patients with actionable genetic abnormalities through off-label drug administration.

**Methods:**

The BELIEVE trial is a platform trial with a single master protocol, conducted under the Clinical Trials Act and the patient-proposed health services (PPHS) scheme. Eligible patients with solid tumors exhibiting actionable alterations were enrolled, and CGP tests covered by national health insurance were employed. Treatment selection, study drugs from collaborating pharmaceutical companies, and treatment schedules adhered to predefined protocols. Primary and secondary endpoints were evaluated, and statistical analysis was conducted based on patient response rates.

**Results:**

The BELIEVE trial offered treatment opportunities for patients with relapse/refractory disease who lacked standard therapies or clinical trial options. This study addresses unmet medical needs and contributes to the establishment of precision medicine systems. Similar trials like NCI-MATCH and TAPUR are being conducted globally. The BELIEVE trial provides a platform for off-label drug administration, collects essential clinical data, and contributes to drug approval applications.

**Conclusion:**

The BELIEVE trial provides hope for patients with actionable genetic abnormalities by facilitating access to targeted therapies through off-label drug administration. It establishes a regulatory framework and promotes collaboration between industry and academia by expanding organ-specific and cross-organ biomarker-based treatments.

## Introduction 

Anticancer drug therapies have advanced with the development of molecular-targeted therapeutics [1]. Precision medicine, which involves the study of genomic abnormalities in cancer and the design of a treatment plan based on the unique characteristics of each patient's cancer, has emerged as a crucial research topic in the field of oncology [1].

Anticancer drugs, including pembrolizumab for the treatment of tumors with high microsatellite instability (mismatch repair deficiency) and high mutation burden, along with pan-TRK inhibitors for NTRK fusion gene, have been demonstrated to be efficacious in the treatment of cross-organ cancers and have received approval for tumor diagnosis in both the United States and Japan [2].

The feasibility of precision medicine is primarily dependent on advancements in genome analysis technology, specifically next-generation sequencing (NGS). Comprehensive genomic profiling (CGP) using NGS can test for dozens to hundreds of genomic abnormalities in cancerous tissue specimens [1]. Thus, cancer genome-based healthcare allows cancer patients to receive genomic medicine treatment. The OncoGuide™ NCC Oncopanel System by Sysmex Corporation and the FoundationOne® CDx Cancer Genomic Profile by Chugai Pharmaceutical were approved and reimbursed under Japan's universal public health care system in 2019. Additionally, the FoundationOne Liquid CDx Cancer Genomic Profile, a CGP test that utilizes circulating tumor DNA in the blood, was approved and reimbursed in August 2021. However, even though CGP detects genetic abnormalities that can be treated by molecular-targeted therapy, patients do not have sufficient on-label access to them.

Off-label drugs are typically not covered under the Japanese universal healthcare system [3], with the exception of when a drug is utilized as part of unique Japanese clinical research, such as registration-directed trials (RDT), advanced medical care (AMC), or patient-proposed health services (PPHS) [4, 5]. Furthermore, ongoing RDTs and AMC trials of off-label drugs are limited [6, 7], and some patients may not meet the eligibility criteria for participation. In contrast to the United States, Japan does not have an expanded access program for individual patients (single patient IND). This current situation in Japan may be a barrier to providing CGP-based precision medicine to patients.

According to Sunami et al. [8], only 13% of patients with potentially actionable alterations, out of a total of 59%, ultimately received appropriate drug treatments. Similar patterns have been observed in other countries, such as the US, Canada, or France [9–11].

Following extensive negotiations with the Ministry of Health, Labour and Welfare (MHLW), the National Cancer Center Hospital (NCCH) launched the BELIEVE trial (NCCH1901; jRCTs031190104), which aims to enhance drug accessibility and collect the clinical data of patients undergoing CGP without an approved indication or corresponding clinical trial.

## Patients and methods

### Study type and ethical considerations

The BELIEVE trial (NCCH1901; jRCTs031190104) was designed as a platform trial using a single master protocol. The objective of this study was to administer off-label drugs for genetic abnormalities and collect efficacy and safety data for patients who were found to have actionable genetic abnormalities, which are genetic abnormalities for which some treatment has been proposed. This study is being conducted under the Clinical Trials Act (Act No. 16 of April 14, 2017) and the PPHS. The PPHS scheme is managed under a mixed billing system, wherein the patient pays the cost of the unapproved/off-label drugs, but routine clinical care is covered under the National Health Insurance System [12].

The study protocol, as well as the informed consent forms, were approved by the National Cancer Center Hospital (NCCH) certified review board and the MHLW as a PPHS. The NCCH was responsible for the overall study coordination, whereas the designated core hospitals for cancer genomic medicine collaborated with medical institutions in the PPHS [13]. The designated core hospitals have sufficient capability to perform genomic testing, conduct genome-based clinical trials, and provide capacity-building programs, including the 12 institutions certified by the MHLW to improve genomic medicine (as of March 2022) [14]. The trial protocol and progress were registered in the Japan Registry of Clinical Trials (jRCTs031190104) [15].

### Eligible patients

Patients with solid tumors showing actionable alterations detected using multiplex gene panel tests were eligible for the trial. The trial included patients with general solid tumors for which standard treatment was completed. Rare cancers with metastasis/recurrence or cancers of unknown primary origin with no standard treatment were included from the time of diagnosis. Most of the cohorts included patients aged ≥ 16 years, while some cohorts included all ages in the study. Patients were included only after obtaining written informed consent for data collection and registering patient information in the Center for Cancer Genomics and Advanced Therapeutics (C-CAT) [16]. Patients who participated in other industry-sponsored clinical trials, investigator-initiated directed clinical trials (IIRDTs), or AMCs were excluded.

Patient enrollment commenced on October 1, 2019, at the National Cancer Center Hospital and has been extended to other designated core hospitals for cancer genomic medicine.

### CGP tests

**Fig. 1:**
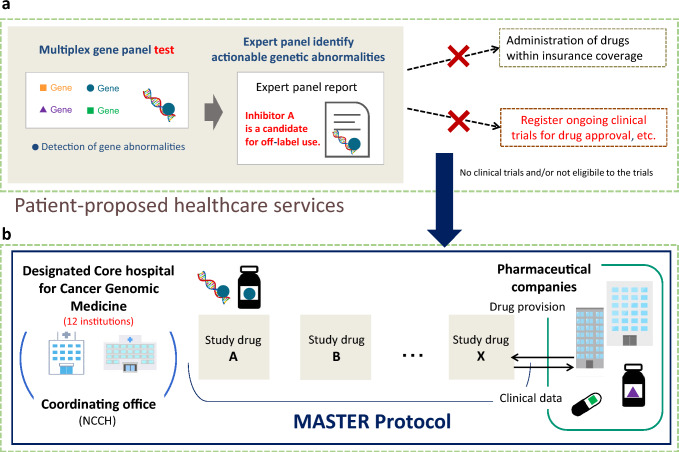
Trial schema of the BELIEVE trial. **a** If a CGP test is performed and some off-label drug is recommended for the patient, the patient would originally be considered for participation in a clinical trial for drug approval already underway. However, if there are no such clinical trials, the patient would be eligible for the BELIEVE trial. **b** Patients would be treated at any of the Designated Cancer Genomic Medicine Core Hospitals if they participate in this trial. Pharmaceutical companies provide the medicines free of charge and will receive efficacy and safety data from the trial

It is a requirement that the CGPs used in this study are covered by the National Health Insurance or are conducted as AMCs (Fig. 1). Two CGPs, namely the OncoGuide™ NCC Oncopanel System (Sysmex Corporation) and the FoundationOne® CDx Cancer Genomic Profile (Chugai Pharmaceutical), are currently covered by the National Health Insurance as CGP tests. Tests performed under the AMC category, including the Todai Onco Panel and Oncomine™ Target Test System, were also eligible for this study (as of September 2022).

### Actionable genetic abnormalities and evidence-level classification

Genetic abnormalities and the corresponding targeted drugs were classified according to the Guidance for Cancer Treatment Based on Gene Panel Testing Using Next-Generation Sequencers (Edition 2.1) issued by three academic societies [17]: the Japanese Society of Medical Oncology, the Japan Society of Clinical Oncology, and the Japan Cancer Association [18]. In this study, an actionable genetic abnormality was defined as a genetic abnormality for which the level of evidence was D or higher (A, B, C, or D), and a molecular tumor board at each institution had determined that the use of the treatment option was appropriate. This Japanese guideline is consistent with the Joint Consensus Recommendation of the Association for Molecular Pathology, American Society of Clinical Oncology, and the College of American Pathologists [19]. A level of evidence of D or higher in the Japanese guideline is equivalent to an evidence level of C or higher in the American recommendations. This level corresponds to evidence level III or higher on the European Society of Medical Oncology (ESMO) scale for clinical actionability of molecular targets (ESCAT) [20]. These are genetic abnormalities for which there is at least one history of treatment based on biomarkers in humans.

### Drug selection for individual patients

The selection of treatment for an actionable genetic abnormality is determined by the investigator or co-investigator on the basis of the findings of the molecular tumor board at the institution. A molecular tumor board examines the obtained genomic data, considers the patient’s background, and uses information like the results of C-CAT reports, which includes potentially applicable drugs and clinical trials. Based on the discussions of the molecular tumor board, the attending physician explains the findings to the patient and ultimately determines a treatment strategy.

### Study drugs

This study included targeted drugs that have already been approved in Japan for some (any) indications and did not include unapproved drugs. In principle, the targeted drugs were not included if a registration-directed trial or an IIRDT was being conducted to extend the indications or if there was a plan to conduct such a trial in the near future.

Collaborating pharmaceutical companies provided the study drugs free of charge. In return, pharmaceutical companies received information collected as part of the study, such as patient background, treatment efficacy, and safety data. We have stated in advance in our IC documents that pharmaceutical companies may use the data from this study for the application of drug approval.

### Treatment schedules and interval evaluation

Treatment dosage and administration of each drug complied with the latest edition of the package insert. Combinations of cytotoxic chemotherapeutic drugs were not permitted for this study. For each drug, delays (i.e., dosing interval prolongation, delaying administration of more than what was prescribed), pauses (i.e., temporary suspension that may resume if certain conditions were met), supportive care, and concomitant use contraindications had to comply with the package insert. Treatment after discontinuation of the protocol was not specifically defined or restricted.

### Study endpoints

The primary endpoint for each drug cohort was the response rate based on the best overall response in patients with measurable lesions for up to 16 weeks after the initiation of treatment. Overall survival, progression-free survival, disease control rate, and incidence of adverse events were also evaluated as secondary endpoints.

### Statistical analysis

The sample size was set to the number of cases that allowed exploratory evaluation and not by a statistical hypothesis. To evaluate efficacy, each drug cohort was expected to include up to 50 patients with measurable lesions. Approximately 30 patients with no measurable lesions were enrolled in the drug cohort.

An interim analysis was performed for each cohort. When ≥ 15 patients were enrolled for each drug cohort, the response rates were assessed based on the Bayesian method to determine whether the drug cohort was worth continuing.

## Results

Table 1 lists the study drugs used as of March 2022. The BELIEVE trial provides treatment opportunities using a matched drug for patients with relapse/refractory disease for whom there is no standard treatment or matched clinical trial or in whom standard therapy has already been administered. Since the only option for such patients was systemic treatment with low levels of evidence, patients were expected to benefit from the addition of new treatment options through their participation in this study.

**Table 1:** List of drugs used in the study (updated March 25, 2022)

No.	Classification	Generic name	Brand name	Provided by^a^	Application in pediatric patients
1	ALK inhibitor	Ceritinib	ZYKADIA tablets 150 mg	Novartis Pharma K.K.	–
2	BCR-ABL tyrosine kinase inhibitor	Imatinib	Glivec tablets 100 mg	Novartis Pharma K.K.	–
3	mTOR inhibitor	Everolimus	AFINITOR tablets 2.5/5 mg AFINITOR dispersible tablets 2/3 mg	Novartis Pharma K.K.	Available
4	BRAF inhibitor	Dabrafenib	Tafinlar capsules 50/75 mg	Novartis Pharma K.K.	–
5	MEK inhibitor	Trametinib	Mekinist tablets 0.5/2 mg	Novartis Pharma K.K.	–
6	VEGF inhibitor	Pazopanib	Votrient tablets 200 mg	Novartis Pharma K.K.	–
7^b^	BRAF inhibitor	Dabrafenib	Tafinlar capsules 50/75 mg	Novartis Pharma K.K.	–
	MEK inhibitor	Trametinib	Mekinist tablets 0.5/2 mg		
8	BCR-ABL tyrosine kinase inhibitor	Nilotinib	Tasigna capsules 50/150/200 mg	Novartis Pharma K.K.	Available
9	JAK inhibitor	Ruxolitinib	JAKAVI tablets 5/10 mg	Novartis Pharma K.K.	–
10	ALK inhibitor	Alectinib	ALECENSA capsules 150 mg	CHUGAI Pharmaceutical Co., LTD	Available
11	Anti-HER2 monoclonal antibody	Trastuzumab	HERCEPTIN for intravenous infusion 150 mg	CHUGAI Pharmaceutical Co., LTD	–
12	Anti-PD-L1 monoclonal antibody	Atezolizumab	TECENTRIQ for intravenous infusion 1200 mg	CHUGAI Pharmaceutical Co., LTD	–
13	TRK inhibitor	Entrectinib	ROZLYTREK capsules 100/200 mg	CHUGAI Pharmaceutical Co., LTD	Available
14	Anti-PD-1 monoclonal antibody	Nivolumab	OPDIVO I.V. infusion 240 mg	ONO Pharmaceutical Co., LTD	–
15^b^	BRAF inhibitor	Encorafenib	BRAFTOVI capsules 50 mg	ONO Pharmaceutical Co., LTD	–
	MEK inhibitor	Binimetinib	MEKTOVI tablets 15 mg	ONO Pharmaceutical Co., LTD	–
16	ALK inhibitor	Crizotinib	XALKORI capsules 200/250 mg	Pfizer Japan Inc	–
17	BCR-ABL tyrosine kinase Inhibitor	Ponatinib	ICLUSIG tablets 15 mg	Otsuka Pharmaceutical Co., Ltd	–
18	Cyclin-dependent kinase 4/6 inhibitor	Abemaciclib	VERZENIO tablets 50/100/150 mg	Eli Lilly and Company	–

*ALK* anaplastic lymphoma kinase, *BCR-ABL* breakpoint cluster region-Abelson, *mTOR* mammalian target of rapamycin, *BRAF* v-raf murine sarcoma viral oncogene homolog B1, *MEK* mitogen-activated protein kinase kinase-extracellular signal-regulated kinase, *VEGF* vascular endothelial growth factor, *JAK* Janus kinase, *HER2* human epidermal growth factor type 2, *PD-1* programmed cell death protein 1, *TRK* tyrosine kinase receptor, *PD-1* programmed cell death 1

^a^All drugs were provided free of charge by pharmaceutical companies

^b^#7 and #15 are combination therapies

## Discussion

The development of drugs with therapeutic effects based on biomarkers, as shown in this study, is extremely important from the viewpoint of satisfying unmet medical needs. Significant progress in genomic medicine in recent years has made it possible to rapidly detect large numbers of genetic abnormalities simultaneously using NGS at an extremely low cost. Biomarker-based clinical research enables the targeting of rare cancers using these technologies, which would lead to the establishment of a system for providing “precision medicine” to individual patients. In Japan, after the CGPs were reimbursed in June 2019, 1,813 patients underwent CGP during the first seven months, compared to only two patients per month before the reimbursement. A total of 7,657 underwent CGP between 15 months and August 2020 [21]. However, of the 747 patients who underwent CGP at designated core hospitals for cancer genomic medicine in the first seven months after reimbursement, only 28 (3.7%) received genomic-matched treatment, which was very low [22]. Since most of the designated core hospitals for cancer genomic medicine participated in the BELIEVE trial, it is expected that more cases will be treated in future.

Similar clinical trials are ongoing in the United States, such as NCI-MATCH and TAPUR (Targeted Agent and Profiling Utilization Registry Trial) [23]. These trials for drug administration with a platform and basket/umbrella design are based on the results of the CGPs. NCI-MATCH (also known as MATCH) is a collaboration between the National Cancer Institute (NCI), a division of the National Institutes of Health, and the ECOG-ACRIN Cancer Research Group, which is a division of the NCI-funded National Clinical Trials Network (NCTN) [23]. TAPUR is a clinical trial conducted by the American Society of Clinical Oncology. Similar to the NCI-MATCH, it is an open-label phase II trial to confirm the efficacy of off-label FDA-approved therapies based on genetic mutations identified by NGS testing [24]. Due to these clinical trials, patients can undergo targeted therapies, although off-label drugs are used. These clinical trials are required in countries other than the United States and Japan. The BELIEVE trial is available under the PPHS scheme, with special MHLW-approved public health insurance and uninsured coverage. The NCI-MATCH is being conducted as a clinical trial, with coverage of evidence development in the United States [25]. Furthermore, it has recently been used for Alzheimer's disease drugs [26].

We need to consider ways in which pediatric patients can benefit from precision medicine. Advances in adult oncology indications have rarely been extended to pediatric cancer treatments. However, extensive research has demonstrated that malignancies in children and adolescents may have the same molecular abnormalities as adult cancers [27–29]. At the beginning of the study, patients aged ≥ 16 years were included, and childhood cancer patients were excluded because the dosage and administration of the study drugs were typically unknown. In April 2021, the protocol was amended to include patients of all ages, thereby allowing pediatric cancer patients to participate in some study drug cohorts.

This study facilitated the collection and analysis of cross-organ, cross-sectional, clinical data for populations carrying each biomarker. The disadvantages of participating in this study included adverse events and the loss of the opportunity to receive sufficient palliative care. Investigators need to be mindful of the condition of patients who have already received several treatments.

Detecting genomic abnormalities using CGP is not the goal for cancer patients; rather, the true benefit can be achieved by providing an opportunity for targeted therapy. We conducted this trial to improve drug access in designated core hospitals for cancer genomic medicine using drugs provided by collaborating pharmaceutical companies. Additionally, this platform trial allowed us to establish a regulatory scheme for drug approval by developing organ-specific and cross-organ biomarker-based treatments and creating a robust infrastructure based on industry–academia collaboration. This study was conducted in accordance with guidelines set forth by the International Conference on Harmonization Good Clinical Practice (ICH-GCP), as opposed to the original Japanese version of the Good Clinical Practice (GCP) guidelines. This is a remarkable limitation of the study.

Historically, clinical trials that deviated from ICH-GCP standards were not accepted in their original form for regulatory approval within Japan. Consequently, there is a strong likelihood that conducting clinical trials based on this platform will necessitate additional trials to secure subsequent approvals.

However, the MHLW has recently clarified that data from specific clinical studies, even if not compliant with the Japanese version of the GCP, can be considered as evidence for regulatory approval. Therefore, it is expected that the related indications will be expanded based on the results of this study.

For cancer genomic medicine to firmly establish itself in Japan, prioritizing concurrent clinical trials for approval alongside other countries holds greater significance than merely augmenting the quantity of drug cohorts within this study. We intend to continue our efforts so that this platform study can serve as a bridge toward ideal cancer genomic medicine in Japan.

## Data Availability

The data analyzed in this study are available from the corresponding author upon reasonable request.
